# CSF biomarkers of neurotoxicity in childhood cancer survivors after cranial radiotherapy or surgery

**DOI:** 10.1002/acn3.52152

**Published:** 2024-07-19

**Authors:** Erik Fernström, Marianne Jarfelt, Malin Blomstrand, Birgitta Lannering, Markus Axelsson, Pontus Wasling, Thomas Björk‐Eriksson, Henrik Zetterberg, Marie Kalm

**Affiliations:** ^1^ Department of Oncology Institute of Clinical Sciences, Sahlgrenska Academy at the University of Gothenburg, Sahlgrenska University Hospital Gothenburg Sweden; ^2^ Department of Pediatrics Institute of Clinical Sciences, Sahlgrenska Academy at the University of Gothenburg Gothenburg Sweden; ^3^ Department of Clinical Neuroscience Institute of Neuroscience and Physiology at the Sahlgrenska Academy, University of Gothenburg Gothenburg Sweden; ^4^ Department of Oncology Institute of Clinical Sciences, Sahlgrenska Academy at the University of Gothenburg Gothenburg Sweden; ^5^ Regional Cancer Centre West Western Sweden Healthcare Region Gothenburg Sweden; ^6^ Department of Psychiatry and Neurochemistry Institute of Neuroscience and Physiology, Sahlgrenska Academy at the University of Gothenburg Mölndal Sweden; ^7^ Clinical Neurochemistry Laboratory Sahlgrenska University Hospital Mölndal Sweden; ^8^ Department of Neurodegenerative Disease UCL Institute of Neurology London UK; ^9^ UK Dementia Research Institute at UCL London UK; ^10^ Hong Kong Center for Neurodegenerative Diseases Clear Water Bay Hong Kong, China; ^11^ Wisconsin Alzheimer's Disease Research Center University of Wisconsin School of Medicine and Public Health, University of Wisconsin‐Madison Madison Wisconsin USA; ^12^ Department of Pharmacology Institute of Neuroscience and Physiology, Sahlgrenska Academy at the University of Gothenburg Gothenburg Sweden

## Abstract

**Objective:**

Treatment of pediatric brain tumors is associated with potential long‐term cognitive sequelae. Patients treated with craniospinal irradiation for posterior fossa tumors are at high risk. New biomarkers that could help to differentiate treatment effects from other causes of cognitive dysfunction would be valuable in tailoring optimal survivorship care. Biomarkers that reflect biological mechanisms behind treatment‐associated cognitive decline would also be important in the evaluation of future treatment regimens for pediatric brain or skull base tumors.

**Methods:**

In this biomarker‐finding study, 10 adult survivors of pediatric medulloblastoma, skull base tumors, and posterior fossa low‐grade glioma underwent study specific lumbar puncture at a minimum of 17 years following treatment. We analyzed cerebrospinal fluid biomarkers reflecting neuron and astrocyte integrity, amyloid metabolism, inflammation, extracellular matrix, synaptic integrity, and blood–brain barrier function. The values were compared with biomarker levels in healthy controls of comparable age.

**Results:**

Biomarkers reflecting neuronal injury (neurofilament light chain protein), astrocyte injury or activation (glial fibrillary acidic protein) as well as inflammation (YKL‐40) were significantly elevated in cancer survivors compared to controls. Biomarkers reflecting amyloid metabolism showed a pattern of decrease in patients treated for medulloblastoma.

**Interpretation:**

The results suggest a potential chronic low‐grade neurodegeneration and astrocyte activation in patients treated for pediatric brain or skull base tumors. Protein biomarkers of CNS disease could potentially be used to increase our understanding of the contribution from different tumor treatments with regard to long‐term symptoms in cancer patients.

## Introduction

Cancer treatment during childhood is associated with a high rate of survival,^1^ but also with complex medical and psychosocial late effects.^2^, ^3^ Of particular concern in the treatment of malignant pediatric brain tumors are potential adverse cognitive effects of cranial radiotherapy. Children with medulloblastoma of the posterior fossa are routinely treated with surgery and craniospinal irradiation (CSI) to the entire central nervous system (CNS) as well as a localized radiation boost to the tumor bed in the posterior fossa, followed by chemotherapy. Cognitive impairment after CSI is common and has been demonstrated in several cognitive domains.^4^, ^5^ However, cognitive impairment after brain tumor treatment can be seen also after partial brain radiotherapy or after surgery alone, as demonstrated by Brinkman et al in a large cohort of adult survivors of pediatric brain tumors assessed by formal neurocognitive testing at a median time of 18 years from initial diagnosis. The group treated with CSI was the most severely impaired across the tested cognitive domains, but cognitive impairment was prevalent also with focal or no radiotherapy. Additional independent risk factors in this study were a history of hydrocephalus or seizures.^6^ Several other risk factors for cognitive impairment have been described, including for example age at treatment.^7^ The incidence of pediatric head and neck tumors is considerably lower than that of brain tumors. There is a knowledge gap about cognitive long‐term sequelae after treatment for head and neck or skull base tumors during childhood, despite the fact that these patients often receive considerable incidental radiation doses to the brain, including the temporal lobes. We initiated a study of adult survivors of pediatric brain and skull base tumors to assess cognitive function, quality of life, and potential biomarkers of late effects in the brain. The results regarding cognitive function and quality of life in survivors of malignant posterior fossa and skull base tumors were recently published and confirmed significant cognitive impairments in patients treated with CSI and a trend toward impaired function also in patients treated for skull base tumors compared to a healthy control group.^8^ We have previously found elevated levels of biomarkers reflecting neuroaxonal injury (neurofilament light chain [NfL] and tau), inflammatory signaling (YKL‐40 [also known as chitinase‐3‐like 1], interleukin [IL]‐15), astrogliosis (glial fibrillary acidic protein [GFAP]), and synapse integrity (GAP‐43 [growth‐associated protein 43]) in the cerebrospinal fluid (CSF) after prophylactic cranial irradiation (PCI) in patients with small cell lung cancer. The study also revealed decreasing levels of soluble amyloid precursor proteins (sAPPα and sAPPβ) and extracellular matrix proteoglycans (brevican and neurocan) up to 1 year after treatment.^9^, ^10^ Based on these previous results, the aim of the present study was to analyze CSF protein biomarkers, possibly reflecting neurotoxicity after cranial radiotherapy or surgery, in a cohort of childhood cancer survivors with long follow‐up time.

## Methods and Materials

### Study protocol and participants

The patient cohort was recruited as part of a larger follow‐up study of adult survivors of childhood brain or skull base tumors. The study was performed within the framework of the long‐term follow‐up clinic for adult childhood cancer survivors at the department of oncology at the Sahlgrenska University Hospital. Three different patient groups were included with the aim of studying long‐term effects of different cranial radiotherapy exposures during childhood: malignant posterior fossa tumors treated with CSI, skull base tumors exposed to incidental brain irradiation and low‐grade astrocytoma of the posterior fossa treated with surgery alone (initially intended as a control group). Additional inclusion criteria were age >18 years and minimum follow‐up time >10 years. Invitations for a visit to the long‐term follow‐up clinic, including study screening, were sent by mail to 38 eligible survivors. Ten individuals did not respond to the letter. Of 28 screened persons, 23 consented to participate in the main study. The entire study protocol included neuropsychological assessment, magnetic resonance imaging (MRI) of the brain, electroencephalography (EEG), patient‐reported outcomes assessment, examination from a speech therapist and physician, endocrine laboratory screening, as well as CSF sampling. Participants could choose to take part in all study modalities or to opt out from certain modalities. The results of the neuropsychological assessment and patient‐reported outcomes for patients with skull base tumors and patients treated with CSI have been published previously.^8^ Of 23 patients included in the main study, 11 patients consented to CSF sampling. One patient treated with CSI had a ventriculo‐peritoneal shunt and had markedly increased CSF protein. This patient subsequently developed symptoms and radiological findings consistent with over‐shunting and was consequently excluded from the statistical analysis. There were no other patients with ventriculo‐peritoneal shunt in the study cohort. Due to the limited number of CSF samples in the study, control CSF was also drawn from an existing biobank of CSF from healthy volunteers. Twelve controls were selected to represent a control group of comparable age and sex distribution. However, due to the paucity of available control samples, no exact age matching was possible. The demographics of the included patients and controls are presented in Table 1. The study was approved by the regional ethics review board (Dnr 721‐15). The collection of control samples was approved in a separate application (Dnr 223‐15). All patients and controls provided written consent.

**Table 1:** Participant characteristics and treatment modalities of patients and controls.

	Controls	All patients	Low‐grade astrocytoma	Skull base tumors	Medulloblastoma
Participant characteristics					
*N*=	12	10	3	4	3
Female (%)	75	40	33	25	67
Age, median (range)	26 (23–36)	32 (27–46)	29 (27–46)	31 (31–33)	34 (31–41)
Age at treatment, median (range)	–	10 (3–15)	8 (3–15)	10 (7–13)	9 (6–11)
Years since treatment, median (range)	–	23 (17–30)	23 (21–30)	22 (17–23)	25 (25–30)
Treatment					
Surgery	–	8/10	3/3	2/4	3/3
Chemotherapy	–	5/10	–	3/4	2/3
Radiotherapy	–	7/10	–	4/4	3/3

### Biomarkers

Biomarkers selected for analysis were proteins involved in maintaining neuronal structural integrity (NfL, tau), astrocyte structural integrity (GFAP), amyloid protein processing (sAPP isoforms alpha and beta, amyloid β 40 and 42 (Aβ40 and Aβ42), and extracellular matrix proteoglycans (brevican). Soluble triggering receptor expressed on myeloid cells 2 (sTREM2) and YKL‐40 was used to investigate microglial activation and neuroinflammation. GAP‐43 and neurogranin were chosen as potential biomarkers of synapse function, integrity, and plasticity. To study potential effects on blood–brain barrier function, we analyzed the levels of the shedded, soluble, form of platelet‐derived growth factor receptor beta (sPDGFRβ).

### Sample analysis

CSF NfL and GFAP concentrations were measured using in‐house enzyme‐linked immunosorbent assays (ELISAs), as previously described.^11^, ^12^ CSF tau, Aβ40, and Aβ42 concentrations were measured using Lumipulse assays (Fujirebio, Ghent, Belgium). sAPPα and sAPPβ concentrations were measured using commercial ELISAs from IBL (Tecan, Männedorf, Switzerland). CSF sTREM2 concentration was measured using an immunoassay with electrochemiluminescence detection, as previously described.^13^ CSF GAP‐43 and neurogranin concentrations were measured using in‐house ELISAs as previously described.^14^, ^15^ CSF YKL‐40 concentration was measured using a commercially available ELISA kit (R&D Systems, Minneapolis, MN, USA). CSF sPDGFRβ concentration was measured by sandwich ELISA (Thermo Fisher Scientific, Waltham, MA, USA). All samples were analyzed as singlicates in a single batch. Intra‐assay coefficients of variation were below 10% for all biomarkers.

### Treatment

Three patients had low‐grade astrocytoma of the posterior fossa and were treated with surgery alone. Patients with tumors of the skull base had sarcomas (*n* = 2), nasopharyngeal cancer (*n* = 1), and angiofibroma (*n* = 1). In this group, all patients had radiotherapy with treatment fields extending into the temporal lobes, brain stem, and cerebellum. Two patients also had surgery, and three patients were treated with chemotherapy. All malignant posterior fossa tumors in the present analysis (*n* = 3) were medulloblastomas and were treated with surgery followed by CSI and a posterior fossa boost. Two of three patients also had chemotherapy. Patients received radiotherapy using either 2D planning techniques or 3D conformal radiotherapy. Patients with tumors of the skull base or nasopharynx were often treated with two opposed lateral fields and one anterior field. The prescribed doses to the primary tumor volume were between 45–61.2 Gy with 1.7–1.8 Gy per fraction. Patients with medulloblastoma received CSI with two opposed lateral fields covering the entire brain and posterior fossa as well as fields covering the entire spinal dural compartment. A sequential boost was delivered to the entire posterior fossa. The range of CSI doses was 32–35 Gy with 1.5–1.75 Gy given as one daily fraction. The total boost dose to the posterior fossa was 53.6–55 Gy.

### Statistics

Descriptive statistics are presented using medians and range or inter‐quartile range. The non‐parametric Mann–Whitney *U* test was used for group comparisons of biomarker values and participant characteristics. Spearman's correlation analysis was used for correlation analyses between biomarkers. R version 3.6.3 (R core team) and SPSS version 27 (IBM Corp.) were used for all analyses.

## Results

**Figure 1:**
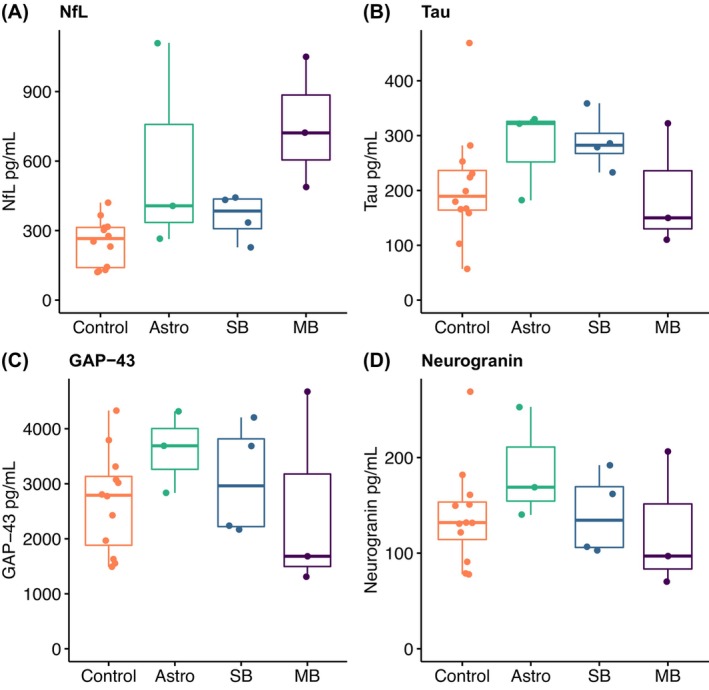
Levels of biomarkers reflecting neuron or synapse degeneration. Astro, patients with low‐grade astrocytoma of the posterior fossa; SB, patients with skull base tumors; MB, patients with medulloblastoma.

**Figure 2:**
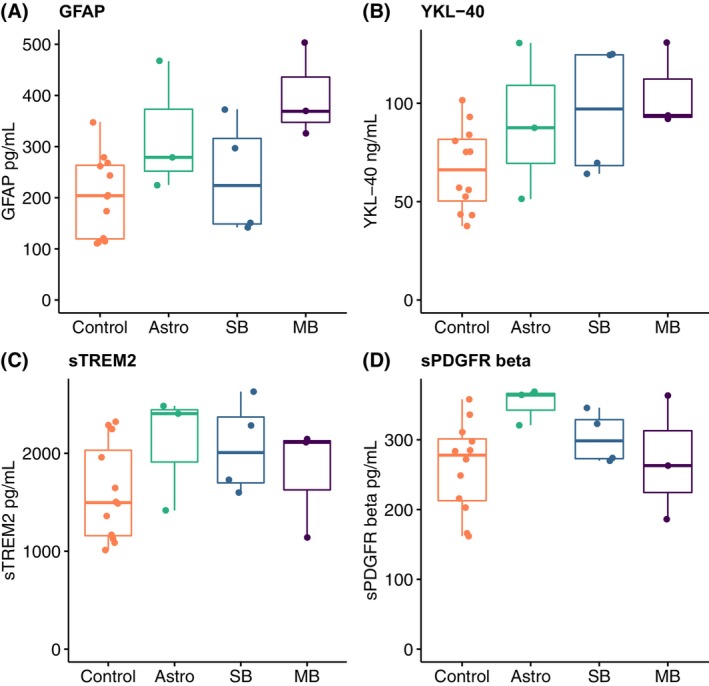
Levels of biomarkers reflecting astrocyte degeneration, inflammation, and blood–brain barrier function. Astro, patients with low‐grade astrocytoma of the posterior fossa; SB, patients with skull base tumors; MB, patients with medulloblastoma.

The median age of the patient group was slightly, but significantly, higher than that of the control group (32 years vs. 26 years, *P* = 0.02, Table 1). The values of all analyzed biomarkers in patients and controls are displayed in Table 2. The patient group had significantly higher levels of NfL compared to controls (Table 2, Fig. 1). Although NfL levels were only modestly increased compared to controls in most patients, three of the adult cancer survivors had NfL values at least 25% above the institutional upper limit of normal using the same assay (<30 years: <380 pg/mL; 30 to <40 years: <560 pg/mL; 40 to <60 years: <890 pg/mL). We also found increased levels of GFAP and YKL‐40 in the treated group (Table 2, Fig. 2). A correlation between age and both NfL (*ρ* = 0.74, *P* < 0.01) and YKL‐40 (*ρ* = 0.66, *P* < 0.01) was observed across the study population. We performed exploratory post hoc analyses in an attempt to reduce the influence of age on the difference between the groups. When removing the three youngest control subjects, the difference in age was no longer significant (32 years vs. 29 years, *P* = 0.11), but NfL was still significantly increased in patients compared to controls (median 439 pg/mL (IQR: 335–722) vs. 304 pg/mL (IQR: 144–317), *P* = 0.01). The difference in YKL‐40 did not remain significant when removing the three youngest controls (median 93 ng/mL (IQR: 70–125) vs. 75 ng/mL (IQR: 56–84), *P* = 0.079). There was no correlation between GFAP and age, neither in the whole study population (*n* = 22, *ρ* = 0.27, *P* = 0.22), nor in the individual groups (patients: *ρ* = −0.13, *P* = 0.73; controls: *ρ* = 0.17, *P* = 0.6). Because of this, no further attempt was made to correct for age in the analysis of GFAP.

**Table 2:** Biomarker levels in all patients and controls.

Biomarker	Controls	Patients	Patients vs controls
	Median (IQR), *N* = 12	Median (IQR), *N* = 10	Mann–Whitney *U*, *P*=
Neuroaxonal injury			
NfL (pg/mL)	266 (137–315)	439 (335–722)	0.003*
tau (pg/mL)	190 (163–242)	283 (182–322)	0.123
Inflammation			
GFAP (pg/mL)	204 (118–265)	312 (225–373)	0.017*
sTREM‐2 (pg/mL)	1496 (1148–2104)	2128 (1600–2406	0.08
YKL‐40 (ng/mL)	66 (48–82)	93 (70–125)	0.025*
Extracellular matrix			
Brevican (ng/mL)	418 (352–467)	489 (304–582)	0.346
Amyloid metabolism			
sAPPα (ng/mL)	316 (246–415)	269 (198–360)	0.456
sAPPβ (ng/mL)	598 (550–758)	542 (359–666)	0.497
Aβ40 (pg/mL)	12130 (9134–14649)	11760 (9675–15488)	1.0
Aβ42 (pg/mL)	1207 (839–1464)	1181 (869–1522)	1.0
Aβ42/Aβ40 ratio	0.098 (0.093–0.1)	0.099 (0.093–0.105)	0.539
Synaptic integrity			
GAP‐43 (pg/mL)	2792 (1799–3194)	3261 (2168–4206)	0.456
Neurogranin (pg/mL)	132 (107–156)	151 (103–192)	0.582
Blood–brain barrier			
sPDGFRβ (pg/mL)	278 (210–305)	322 (270–363)	0.107
Albumin ratio	–	4.8 (3.2–8.5)	–

IQR, inter‐quartile range.

* *P* < 0.05.

**Figure 3:**
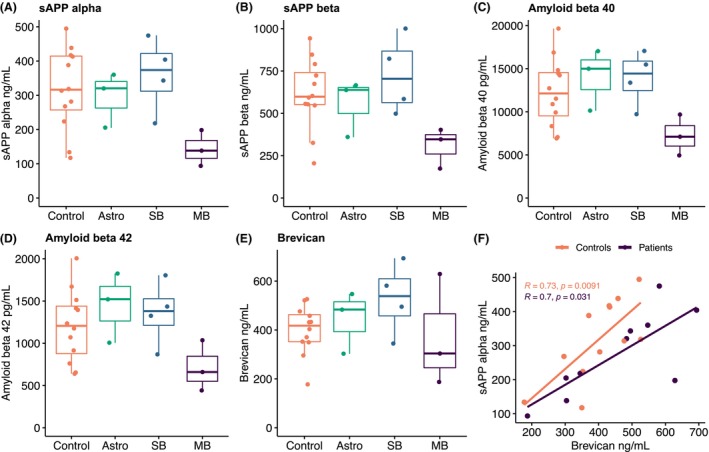
(A–E) Levels of biomarkers reflecting amyloid metabolism and extracellular matrix integrity. (F) Correlation between sAPPα and brevican. Correlation coefficients represent Spearman's rho. Astro, patients with low‐grade astrocytoma of the posterior fossa; SB, patients with skull base tumors; MB, patients with medulloblastoma.

Biomarkers reflecting amyloid metabolism (sAPPα, sAPPβ, Aβ40, and Aβ42) showed no significant difference between the control and patient groups (Table 2). Patients with astrocytoma and skull base tumors had values that qualitatively resembled controls. However, in the patients treated with CSI, a tendency was seen toward decreased values compared to controls (Fig. 3). The Aβ42/Aβ40 ratio was very similar between patients and controls (Table 2). We found no significant difference between patients and controls in the biomarkers tau, GAP‐43, neurogranin, sPDGFRβ, or brevican. We observed a moderate to strong correlation between each and all of the biomarkers of synapse integrity (GAP‐43, neurogranin), amyloid metabolism (Aβ40, Aβ42, sAPPα, sAPPβ), and extracellular matrix (brevican) across the entire study population (Table S1, Fig. 3F).

## Discussion

The aim of this biomarker‐finding study was to investigate CSF biomarkers reflecting CNS injury after cancer treatment given during childhood in a cohort with long follow‐up time. Due to the long follow‐up and the need for lumbar puncture, the sample size was small. Nevertheless, we found modestly elevated NfL values in patients compared to controls, with numerically higher values in patients treated with CSI. In addition, biomarkers of astrocyte activation/degradation (GFAP) as well as inflammatory signaling (YKL‐40) were modestly increased in patients compared to controls and this was especially pronounced in patients treated with CSI. Due to the small and heterogenous study cohort, it was not possible to draw conclusions regarding the role of single treatment modalities or the relationship between CSF proteins and cognitive outcomes. However, the inclusion of a healthy control group adds information about the effect sizes of these biomarkers, something which can be of value when designing future studies of late effects after treatment for pediatric brain tumors.

NfL is an abundant structural component of the axonal cytoskeleton and is released into the CSF as a consequence of axonal injury, regardless of etiology.^16^ CSF levels of NfL are associated with disease activity in several neurologic disorders, including multiple sclerosis,^17^ ALS,^18^ and Alzheimer's disease.^19^ Recent methodological advances have led to development of assays that allow the quantification of NfL in serum or plasma.^20^ NfL in blood is quickly gaining acceptance as a biomarker reflecting neuroaxonal injury without the need for lumbar puncture.^21^ This opens new possibilities to study larger cohorts of adult cancer survivors, where NfL could be more readily studied in relation to cognitive outcomes and different treatment modalities.

Preclinical evidence strongly suggests that signaling from activated microglia play an important role in inducing a neurotoxic, pro‐inflammatory phenotype in astrocytes following CNS injury and disease.^22^ TREM2 (triggering receptor expressed on myeloid cells 2) is an immune receptor expressed by microglia, and its shedded soluble form (sTREM2) has been investigated as a potential biomarker of microglia activity in neurological disease.^23^ We found numerically elevated levels in this cohort, but the comparison with healthy controls did not reach statistical significance. GFAP, a type III intermediate filament, is highly expressed in reactive astrocytes in areas of reactive gliosis.^24^ In CSF, it is regarded as a biomarker of astrocyte injury/activation and is elevated in several neurologic diseases, including MS^25^ and neurodegenerative dementias.^26^ YKL‐40 is a glycoprotein secreted by various cell types. Its physiological role has not been firmly established but it is considered to play a role in tissue remodeling during inflammation.^27^ YKL‐40 is highly expressed in reactive astrocytes in brain tissue from patients with Creutzfeldt‐Jakob disease and Alzheimer's disease^28^, ^29^ and the CSF levels of YKL‐40 have been found to be elevated in several neurologic diseases with a neuroinflammatory component.^28^, ^30^, ^31^ The moderately elevated levels of GFAP and YKL‐40 observed in cancer survivors in the present study could potentially reflect both long‐term reactive gliosis and ongoing low‐grade neuroinflammation.

In our previous longitudinal study of adult small cell lung cancer patients receiving PCI, several biomarkers, including NfL, GFAP, and YKL‐40, were transiently elevated 3 months following cranial irradiation, indicating an acute injury to neurons and astrocytic cell populations as well as an inflammatory response to radiotherapy.^9^ Interpreting biomarkers in patients treated for primary brain tumors is more complex. As is the case with NfL, recently developed ultra‐sensitive assays allow quantification of GFAP in peripheral blood, opening up possibilities to study astrocyte injury and activation in cancer patients, both during treatment and at long‐term follow‐up. In a recent study of blood‐based biomarkers in patients undergoing surgery and postoperative radiotherapy for malignant glioma, plasma NfL and GFAP were both correlated to preoperative tumor volume. There was a postoperative increase in NfL but both GFAP and NfL subsequently decreased during and up to 4–8 weeks after radiotherapy.^32^ It is possible that any immediate effects from radiotherapy on biomarker levels could have been masked by effects of intracranial surgery as well as GFAP expressed by the tumor tissue itself. This also illustrates that biomarker levels will be influenced by both tumor‐, patient‐, and treatment‐related factors.

In the previous study of adult patients undergoing PCI, we found decreasing levels of biomarkers of amyloid metabolism. The reduction in these biomarkers occurred already at 3 months but the levels remained decreased 1 year after radiotherapy and were also correlated with a reduction in extracellular matrix biomarkers.^9^, ^10^ During intracellular trafficking, membrane‐bound APP is cleaved by α‐secretase into sAPPα, which may serve a physiological role in promoting neuronal plasticity and survival.^33^ The less common alternative cleavage by β‐secretase generates sAPPβ, and the remaining membrane‐bound peptide can then be sequentially cleaved by γ‐secretases into amyloid β fragments of various length, including amyloid β ending at residue 42 (Aβ42), which has the potential to form insoluble plaques in Alzheimer's disease and Down's syndrome.^34^ A reduction in CSF Aβ42 is one of the biomarker hallmarks of Alzheimer's disease^35^ and correlates with the deposition of amyloid in plaques in the cortex.^36^ However, reduced levels of CSF Aβ42 have also been found in diseases without plaque formation,^37^, ^38^ suggesting that decreased levels of Aβ in CSF may reflect amyloid dysmetabolism of different aetiologies. The reduction and correlation of both sAPPα and sAPPβ as well as Aβ observed in patients treated with CSI in the present study would perhaps suggest alterations in amyloid metabolism up‐stream of the cleavage of APP into sAPPα and sAPPβ. Although the group treated with CSI was too small for formal statistical comparison, the reduced levels of amyloid biomarkers are in line with the results seen in patients treated with PCI,^9^, ^10^ warranting further study of the effects of cranial radiotherapy on amyloid metabolism in the context of radiation toxicity.

The major limitation of this pilot study is the small sample size. The patient group was heterogenous, and the need for lumbar puncture made the accrual of participants difficult. This also meant that the age was not perfectly matched between patients and controls. Although cranial radiotherapy is an established risk factor for cognitive decline, intracranial surgery as well as the administration of chemotherapy could also have influenced the results of the present study.^39^

## Conclusions

The data from this pilot study suggest that protein biomarkers of CNS disease could potentially be used to increase our understanding of the contribution from different tumor treatments with regard to long‐term symptoms in cancer patients. These data also suggest a potential ongoing chronic low‐grade neurodegeneration, as well as astrocyte activation or degradation, many years after treatment for pediatric brain or head and neck tumors. With the advent of assays that can detect nervous system specific biomarkers also in blood, future studies will be able to assess these biomarkers in larger cohorts of cancer survivors.

## Author Contributions

Conception and design: EF, MJ, MB, BL, TB‐E, HZ, and MK. Acquisition, analysis, or interpretation of data: All authors. Statistical analyses: EF. Drafting of the manuscript: EF, MK, and MB. Critical revision of the manuscript for important intellectual content: All authors.

## Conflicts of interest

HZ has served at scientific advisory boards and/or as a consultant for Abbvie, Acumen, Alector, Alzinova, ALZPath, Amylyx, Annexon, Apellis, Artery Therapeutics, AZTherapies, Cognito Therapeutics, CogRx, Denali, Eisai, Merry Life, Nervgen, Novo Nordisk, Optoceutics, Passage Bio, Pinteon Therapeutics, Prothena, Red Abbey Labs, reMYND, Roche, Samumed, Siemens Healthineers, Triplet Therapeutics, and Wave, has given lectures in symposia sponsored by Alzecure, Biogen, Cellectricon, Fujirebio, Lilly, Novo Nordisk, and Roche, and is a co‐founder of Brain Biomarker Solutions in Gothenburg AB (BBS), which is a part of the GU Ventures Incubator Program (outside submitted work). MK is an employee at AstraZeneca.

## Supporting information


**Table S1.** Spearman's correlation analysis between all included biomarkers of amyloid metabolism, synapse‐ and extracellular matrix integrity in all study participants.

## Data Availability

The data that support the findings of this study are available on request from the corresponding author. The data are not publicly available due to privacy or ethical restrictions.
