# Supplementary material for: CSF biomarkers of neurotoxicity in childhood cancer survivors after cranial radiotherapy or surgery
Source: Ann Clin Transl Neurol. 2024 Jul 19;11(9):2382–91. doi: 10.1002/acn3.52152 (PMC11537133; doi:10.1002/acn3.52152)
Supplement: Supplementary file 1 — Table S1. Spearman's correlation analysis between all included biomarkers of amyloid metabolism, synapse‐ and extracellular matrix integrity in all study participants. [file ACN3-11-2382-s001.docx]

| SUPPLEMENTARY TABLE 1 Spearman’s correlation analysis between all included biomarkers of amyloid metabolism, synapse- and extracellular matrix integrity in all study participants. | | | | | | | | |
| --- | --- | --- | --- | --- | --- | --- | --- | --- |
|  |  |  | brevican | Amyloid β 40 | Amyloid β 42 | sAPPα | sAPPβ | neurogranin |
| Spearman's rho | Amyloid β 40 | Correlation Coefficient | .652^**^ |  |  |  |  |  |
|  |  | Sig. (2-tailed) | 0.001 |  |  |  |  |  |
|  |  | N | 22 |  |  |  |  |  |
|  | Amyloid β 42 | Correlation Coefficient | .683^**^ | .986^**^ |  |  |  |  |
|  |  | Sig. (2-tailed) | <0.001 | <0.001 |  |  |  |  |
|  |  | N | 22 | 22 |  |  |  |  |
|  | sAPPα | Correlation Coefficient | .635^**^ | .827^**^ | .790^**^ |  |  |  |
|  |  | Sig. (2-tailed) | 0.001 | <0.001 | <0.001 |  |  |  |
|  |  | N | 22 | 22 | 22 |  |  |  |
|  | sAPPβ | Correlation Coefficient | .639^**^ | .744^**^ | .698^**^ | .950^**^ |  |  |
|  |  | Sig. (2-tailed) | 0.001 | <0.001 | <0.001 | <0.001 |  |  |
|  |  | N | 22 | 22 | 22 | 22 |  |  |
|  | neurogranin | Correlation Coefficient | .666^**^ | .805^**^ | .853^**^ | .516^*^ | .443^*^ |  |
|  |  | Sig. (2-tailed) | 0.001 | <0.001 | <0.001 | 0.014 | 0.039 |  |
|  |  | N | 22 | 22 | 22 | 22 | 22 |  |
|  | GAP43 | Correlation Coefficient | .695^**^ | .792^**^ | .839^**^ | .531^*^ | .479^*^ | .955^**^ |
|  |  | Sig. (2-tailed) | <0.001 | <0.001 | <0.001 | 0.011 | 0.024 | <0.001 |
|  |  | N | 22 | 22 | 22 | 22 | 22 | 22 |
| **. Correlation is significant at the 0.01 level (2-tailed). | | | | | | | | |
| *. Correlation is significant at the 0.05 level (2-tailed). | | | | | | | | |
